# Academic motivation among senior students majoring in rehabilitation related professions in China

**DOI:** 10.1186/s12909-021-03016-9

**Published:** 2021-11-17

**Authors:** Huiling Hu, Hongmei Luo

**Affiliations:** 1 Department of Rehabilitation Medicine, The Second Xiangya Hospital of Central South University, Renmin Middle Road NO.139, Furong district, Changsha, Hunan China; 2 Department of Rehabilitation Medicine, Tongji Hospital, Tongji Medical College, Huazhong University of Science and Technology, Wuhan, China

**Keywords:** Academic motivation, Medical education, Motivation, Rehabilitation education, Rehabilitation students

## Abstract

**Objective/background:**

In mainland China, most universities offer general rehabilitation curricula rather than specialized curricula. The purpose of the current study is to investigate senior students’ academic motivation for rehabilitation and examine whether it varies among different curriculum structures, students’ gender, specific interests, and parental average education level.

**Methods:**

This cross-sectional study recruited both senior students in general and those who specialized in rehabilitation curricula using an online survey. The Academic Motivation Scale (AMS) was used to measure academic motivation.

**Results:**

The response rate was 74.68%, and 59 senior students in total (male: 34.48%; female: 65.52%) were analyzed. Twenty-nine (50.00%) students were from a general rehabilitation curriculum at Guangxi Medical University, and the rest (*n* = 29, 50.00%) were from a specialized curriculum at West China Medical School of Sichuan University. The overall average academic motivation score was 30.96 ± 5.92. Students in the specialized rehabilitation curriculum (32.85 ± 6.26) showed a significantly higher academic motivation score than those in the general rehabilitation curriculum (29.10 ± 5.00, *p*<0.05). Male (31.13 ± 5.67) and female (30.88 ± 6.12) students had equally high scores (*p* = 0.88). Students who had specific interests (29.81 ± 4.73) and those who did not (24.69 ± 4.92) shared the same academic motivation (t = 2.00, *p* = 0.06).

**Conclusions:**

Senior rehabilitation science students in specialized curricula have higher levels of academic motivation than those in general curricula. There was no significant difference in academic motivation scores based on students’ gender, specific interests, or parental average education levels.

**Supplementary Information:**

The online version contains supplementary material available at 10.1186/s12909-021-03016-9.

## Introduction

### Rehabilitation education in China

Currently, China’s educational training in rehabilitation is still in the developing stage. The academic standards of most rehabilitation curricula in China are not able to meet the minimum requirements proposed by the World Federation of Occupational Therapists (WFOT), the World Confederation for Physical Therapy (WCPT), or other international therapist federations [1]. High academic motivation is a critical component of successful learning performance and scholarly achievement [2]. China has been left behind in regard to carrying out regular rehabilitation education, such as undergraduate rehabilitation and postgraduate education [3]. Undergraduate rehabilitation education did not start until 2001, and the related college education (diploma only) has only been implemented for just over 20 years [4]. The education system of rehabilitation is not perfect, and the training methods and quality are quite different from those found in other countries. Most universities still provide the following two curricula: rehabilitation therapy science (bachelor’s degrees) or rehabilitation technology (diploma only), such as Hunan University of Tradition Chinese Medicine and Guangxi Medical University. Both are general professional curricula with a comprehensive curriculum structure. Only a few colleges and universities have a rehabilitation therapy science curricula with a specific curriculum structure of physiotherapy or occupational therapy, such as Kunming Medical University and West China Medical School of Sichuan University [5].

Additionally, rehabilitation is not the first choice for a number of students. It is a universal phenomenon in China that some students in the rehabilitation profession also apply for other majors, but they often fail to enroll in the others. These students have poor recognition of the major and show a low level of professional identity [6]. The complex circumstances make academic motivation among Chinese students majoring in rehabilitation more complicated.

### Academic motivation

Motivation allows learners to participate in learning activities and maintain their learning status [7, 8]. Academic motivation is a student’s desire (as reflected in his or her approach, persistence, and level of interest) regarding academic subjects when the student’s competence is judged against a standard of performance or excellence [9]. This concept will affect the deep processing of investment information. Additionally, it relates to self-efficacy, goal setting, and the generation of achievement expectations [10]. During the past 30 years, motivation has been a high-frequency word in the field of educational psychology. Some articles have pointed out that academic motivation does not seem to be a valid predictor of grade point average (GPA) [11]. However, there are still immense benefits of motivation because it is helpful with numerous learning outcomes directly relating to academic achievement [12], along with positive self-concept [13] and persistence [14]. There are significant consequences resulting from motivation, such as academic performance and scholarly achievement, intention to drop out, and absenteeism [2]. Of the three kinds of motivation (intrinsic motivation, extrinsic motivation, and amotivation), intrinsic motivation is considered more significant in education. This factor can make a better change in regard to promoting efficient learning and creativity. There are three subscales of intrinsic motivation, to know, toward accomplishment and to experience stimulation. Intrinsic motivation leads people to perform activities with internal emotions [15]. For example, people will feel a sense of joy, curiosity, happiness, interest, or other internal emotions. However, extrinsic motivation makes people keep the result in mind, regardless of the outcome is. The result can be a positive award, or it can also be a negative penalty [16]. There are also three subfields of extrinsic motivation; identified, introjected and external regulation. Amotivation occurs when a student has no intention to display the behavior [15]. If the student does not display academic behavior voluntarily and there is no reason why the behavior should be displayed, then it is considered that the student feels amotivated [17]. Different results correspond to each type of academic motivation; thus, motivation can predict students’ academic achievements and learning performance, participation and learning attitude, and the ability to stick to a learning goal [18].

It is advised to develop academic motivation to guide students to cultivate a habit of deep learning and obtain a good grade, especially for health care-related professions students [19].

### Potential influential factors of academic motivation

Evidence shows a list of the potential influencing factors of academic motivation, including students’ gender, specific interests, and parental average education level. Overall, these factors make up over 20% of the difference in academic motivation [20]. In addition, Lyndon and his colleagues [21] suggested that curriculum structures can also affect students’ motivation. To improve students’ academic performance and psychological status, researchers have examined these motivational profiles. They have indicated that faculty and staff in school could adjust educational activities, teaching strategies of regulation and curriculum structures to make students more self-determined [22].

However, no research has focused on academic motivation and associated factors for students in the rehabilitation-related profession in China. Thus, the purpose of the current study is to investigate rehabilitation students’ academic motivation in China. Based on the above previous research, we want to examine whether differences exist in students’ academic motivation among students’ gender, specific interests, parental average education level, and curriculum structures. The following are our hypotheses: male and female students share the same academic motivation; students who have specific interests have higher academic motivation; students whose parental average education level is higher have higher academic motivation; students who major in specialized have higher academic motivation general curriculum students.

## Method

### Design and participants

We used a cross-sectional descriptive survey carried out between October and November of 2020. The target population for this study consisted of senior students majoring in rehabilitation-related professions. Through 3 years of learning in their curricula, senior students have already acquired a fundamental and relatively comprehensive understanding of their major and have benefited from the different curriculum structures. Researchers obtained a convenience sample by taking a cross-section of senior students at two universities, namely, Guangxi Medical University and West China Medical School of Sichuan University. Guangxi Medical University was selected because the researcher graduated from there, and the school offers a set of generalized rehabilitation curricula for a bachelor’s degree in science of rehabilitation. West China Medical School of Sichuan University was selected because it provides specialized PT (physical therapy) or OT (occupational therapy) curricula. It was the first university to recruit rehabilitation profession students in 1997, and it subdivided the general profession into two more specialized curricula, namely, PT and OT, in 2008. These two curricula settings were accredited by the WCPT and WFOT in 2013 and 2014, respectively [23].

### Instruments

In this study, academic motivation was measured by the Academic Motivation Scale. The survey was conducted online using Wenjuanxing, an online survey management tool, via WeChat. It is convenient to communicate and share questionnaires through WeChat in China. The survey was divided into three sections: participants’ informed consent, demographic information including gender, grade, the full name of the profession, parental educational level, the subfield of the profession, and specific interests (only for general curriculum students), and the Academic Motivation Scale.

The Academic Motivation Scale (AMS) is most widely used as an assessment instrument in academic motivation [22]. It contains 28 items rated on a 7-point Likert scale. The AMS is rooted in the self-determination theory, a theory which focuses on the approach to human motivation and personality [24]. This theory applies traditional empirical method and organismic metatheory to explore the psychological mechanisms relating to inner resources for personality and behavioral self-regulation [17]. Supported by the self-determination theory, the AMS is a valid and reliable way to assess students’ motivation and now has been applied in recent decades in high school and college education [22]. .The college version of the AMS has been used to assess university students’ intrinsic motivation, extrinsic motivation, and amotivation toward education. AMS appears to be among the most popular academic motivation measures for college students [25]. The scale’s developers have contended that the AMS is cross-culturally valid [26]. The AMS in this study was adapted from the Chinese translated version by Zhang, Li, Li, Li & Zhang [2].

### Procedures

After the Creighton University Institutional Review Board approved this proposal, coordinated faculty from Guangxi Medical University and Sichuan University helped distribute the questionnaires. They sent the questionnaire link to the senior students via WeChat. Before the participants were allowed access to the survey, they had to acknowledge their consent by reading the consent information on the top of the online survey link. Only when all the items on the questionnaire were answered could it be considered a valid response. The survey was kept anonymous, and 4 weeks were provided for survey completion. The coordinated faculty reminded the participants about the deadline.

### Data analysis

To answer the research questions, we used simple descriptive statistics, frequency testing and hypothesis testing to analyze demographics collected from the survey. For differences in academic motivation based on the profession’s curriculum structures, a specific interest in the subfield of rehabilitation, and gender, we use independent samples t-test. For differences in academic motivation based on parental educational level, we used one-way analysis of variance (ANOVA). The significance level was set at 0.05.

## Results

We received 59 responses from 79 (74.68%) selected senior students from a total of two universities. Twenty-nine out of 31 (93.55%) students at Sichuan University responded to the questionnaire, and all responses were valid for analysis. Thirty out of 48 (62.50%) students at Guangxi Medical University responded to this questionnaire. However, one response was invalid because the participant all chose the first answer option for 28 question items and finished the questionnaire within a much shorter time than average. Thus, a total of 58 responses were used for data analysis.

Table 1 shows the demographic information of the 58 valid responses. Exactly half of these participants were from the general rehabilitation science curriculum at Guangxi Medical University. The other half were from the rehabilitation curriculum with a specialized subfield at Sichuan University (*n* = 29, 50%). As the table shows, the higher education level, the fewer parents.

**Table 1:** Demographics of the valid responded senior students

Items	Categories	N	Percentage (%)
**Gender**	Male	20	34.48
	Female	38	65.52
**Your profession**	Rehabilitation (General)	29	50.00
	Rehabilitation (PT)	22	37.93
	Rehabilitation (OT)	7	12.07
**Your parents’ education level (the highest)**	Primary education or lower	11	18.97
	Secondary education	27	46.55
	College education	11	18.97
	Undergraduate education	8	13.79
	Postgraduate education or higher	1	1.72
**Regions of university**	Sichuan	29	50.00
	Guangxi	29	50.00

Table 2 shows the subfields of interest of the senior students majoring in rehabilitation science (general). Most of these senior students cultivated an interest in at least one subfield (*n* = 25, 86.21%). Only a few did not have a specific interest, and they did not care which rehabilitation subfields they worked on in the future (*n* = 4, 13.79%).

**Table 2:** Demographics of rehabilitation (general) senior students’ subfield of interest

Categories	N	Percentage (%)
Physical therapy	10	40.00
Pediatric	5	20.00
Respiratory therapy	3	12.00
Speech therapy	3	12.00
Traditional Chinese Medicine	1	4.00
Sports injury	1	4.00
Occupational therapy	1	4.00
Post-natal rehabilitation	1	4.00

Table 3 shows the distributions of the seven subscales of the AMS. All the subscales of the AMS have high mean scores except for the subscale of amotivation. Senior students from two kinds of curricula had good academic motivation with an average score of 30.96.

**Table 3:** The detailed information of the subfield of AMS

	Max	Min	Mean	Std. Dev	95% CI	
					Lower bound	Upper bound
**Intrinsic motivation-to know**	7.00	4.00	5.65	0.96	5.39	5.90
**Intrinsic motivation-toward accomplishment**	7.00	2.75	5.48	1.14	5.18	2.78
**Intrinsic motivation-to experience stimulation**	7.00	3.50	5.56	1.00	5.30	5.82
**Extrinsic motivation- identified**	7.00	3.50	5.75	0.96	5.50	6.00
**Extrinsic motivation-introjected**	7.00	1.75	4.96	1.29	4.62	5.30
**Extrinsic motivation- external regulation**	7.00	3.75	5.78	0.97	5.52	6.03
**Amotivation**	6.00	1.00	2.21	1.27	1.87	2.54
**Total points**	41.00	18.50	30.96	5.92	29.40	32.52

Each rating scale is 1-7

Table 4 shows that, on average, students from the specialized rehabilitation curriculum had significantly higher academic motivation scores (32.85 ± 6.26) than those from a general rehabilitation curriculum (29.10 ± 5.00) (t = 2.50, *p* = 0.02). However, from the table the difference based on parental education levels was not significant (F(3,54) = 2.48, *p* = 0.07). Similarly, there was no significant difference between males (31.13 ± 5.67) and females (30.88 ± 6.12) (t = 0.15, *p* = 0.88). There was also no significant difference based on whether the senior students had specific interests (29.81 ± 4.73) or not(24.69 ± 4.92) (t = 2.00, *p* = 0.06). For further study, we analyzed the three different subfields of the AMS between the specialized and general curriculum students. The analysis showed that specialized curriculum students not only had higher levels of intrinsic motivation (17.62 ± 3.26) than did general curriculum students (15.76 ± 2.39) (t = 2.48,*p* = 0.02) but also had higher levels of extrinsic motivation (17.48 ± 3.10) than did those from general the rehabilitation curriculum (15.47 ± 2.10) (t = 2.89, *p* = 0.01). However, they had a similar level of amotivation (t = 0.46, *p* = 0.65).

**Table 4:** AMS difference

	Frequency (n)	Mean	Std. Dev	95% CI		*P*
				Lower bound	Upper bound	
**Gender**						0.88 ^a^
Male	20	31.13	5.67	28.48	33.78	
Female	38	30.88	6.12	28.86	32.89	
**Curriculum structure**						0.02^a^*
Specialized	29	32.82	6.26	30.43	35.20	
General	29	29.10	5.00	27.20	31.01	
**Parental education**						0.07^b^
Elementary school	11	27.61	4.65	24.49	30.73	
Middle and high school	27	32.10	5.23	30.03	34.17	
College	11	29.43	7.21	24.59	34.27	
University graduate or higher	9	33.50	6.21	28.72	38.28	
**Have specific interests or not**						0.06 ^a^
Yes	25	29.81	4.73	27.86	31.76	
No	4	24.69	4.92	16.86	32.52	
**Intrinsic motivation**						0.02^a^*
Specialized	29	17.62	3.26	16.38	18.86	
General	29	15.76	2.39	14.85	16.67	
**Extrinsic motivation**						0.01^a^*
Specialized	29	17.48	3.10	16.31	18.66	
General	29	15.47	2.10	14.68	16.27	
**Amotivation**						0.65^a^
Specialized	29	2.28	1.45	1.73	2.84	
General	29	2.12	1.10	1.71	2.54	

^a^independent samples t-test

^b^one-way analysis of variance

^*^significant difference

## Discussion

Although the AMS has been widely used in western counties, little research discusses academic motivation and associated factors for rehabilitation-related profession students in China. Our results show that students enrolled in a specialized rehabilitation curriculum have higher academic motivation scores than those of general rehabilitation curriculum students. No difference was found based on students’ gender and their parental education level.

The source of motivation is different, and the impact may be individualized [27]. Specifically, the evidence shows that students’ academic motivation varies due to the curriculum structures [21]. Our study supports this view, and we find that specialized curriculum students have higher levels of academic motivation. We consider this outcome to be related to the accreditation of the curriculum settings. The WFOT and the WCPT have detailed curriculum requirements, including content, teaching, learning and assessment strategies, and skill development for educational curricula. The WCPT has accredited six curricula providing a bachelor’s degree in physical therapy or a Bachelor of Science (BSc) in rehabilitation therapy (Physical Therapy) in China [28]. Similarly, the WFOT has approved six curricula providing a Bachelor of Occupational Therapy or a Bachelor of Science in occupational therapy in China [29]. One of our survey sites, the West China Medical School of Sichuan University, received accreditation from both groups. According to the education minimum standards and the guidelines of WFOT and the WCPT, autonomous learning and lifelong learning are emphasized, such as “Graduates should be autonomous learners with developed lifelong learning skills and an ability to engage in continuing professional development” [30, 31]. We think these criteria may lead to the fact that students in West China Medical School of Sichuan University have higher intrinsic academic motivation than those from Guangxi Medical University. The Academy of Guangxi Medical University has also reflected on their curriculum setting and has been trying to refer to the educational requirements of the WCPT and the WFOT to provide specialized rehabilitation curricula in the future [32]. Specifically, we found that differences are significant in the subscales of intrinsic motivation (to-know and to-experience stimulation) and extrinsic motivation (identified and external regulation). Students at West China Medical University of Sichuan University have better intrinsic motivation (to-know and to-experience), so they have more pleasure, satisfaction, and stimulating sensations of experience while learning. This study finding is consistent with those of a previous study [33]. Similar to students in international business professionals, senior students at West China Medical University of Sichuan University value external regulations, as they want to give a more positive image to family and friends [34]. Similarly, we found students in West China Medical School of Sichuan University have higher extrinsic motivation scores on the identified and external regulation subscales. Stated another way, these students tend to value their study’s significance and consciously accept the regulatory process [35]. Most likely, they benefit from small class teaching models, and each of them has one senior undergraduate tutor [23]. Interestingly, the latest research reveals that virtual gamification enhances intrinsic motivation, which is a potential application in future online learning [36].

There are multitudinous factors but students’ perspective, gender, and the average education level of their parents are considered the most critical predictors of academic motivation [20]. Other factors include students’ family support, such as family socioeconomic status and parental expectations [37]. Female students seem to outperform males in many aspects of academic achievement, and gender differences in motivation are considered a part of the reason why [38]. However, another study reported that male students have higher motivation levels than female students [39]. Our study found no significant difference in academic motivation based on students’ gender or their parental education level. These findings are different from those of previous studies. We may ascribe the differences to the regional and time disparities. Currently, we emphasize more than ever the education equality of men and women. Factors including gender bias and parental educational level may not affect students’ academic motivation so much. Another potential reason is that our sample size is insufficient.

We consider generalizing our results among students studying in general and specialized rehabilitation curricula in China. There are only six occupational therapy and physiotherapy curricula accredited by international organizations thus far. Academic motivation varies based on curriculum structure among students majoring in rehabilitation science. It is recommended that educators arrange specific curricula to enhance medical students’ motivation for research [40]. For rehabilitation students, educators may also consider transforming the curriculum structure from the general rehabilitation curriculum to a specialized one in the rehabilitation science field’s undergraduate education. Considering this point further, educators may deliberate the improvement of not only the curriculum structure but also classroom teaching and examinations to help students have both better intrinsic and external motivation [24].

### Limitations and recommendations

The academic motivation scale we used has been found to be cross-culturally valid in many education professions; nevertheless, it has not been applied among rehabilitation students. Therefore, it is necessary to validate this scale in the further study.

Moreover, our survey was conducted in two different universities; thus, there may be regional and university differences. For example, the West China Medicine School of Sichuan University recruits students who receive higher grades on their college entrance examination compared to those at Guangxi Medical University. This may affect the result and lead the students at Sichuan University to have higher academic motivation scores.

Additionally, the low number of responses received from Guangxi Medical University practitioners may result in a sample bias. The participants in our study were all senior students who were struggling during their clinical practicum period. They already have a hectic schedule in their last academic year. On the other hand, the questionnaires we used were self-assessments. Thus, if the participants were engaged in their work these days, the 28-item questionnaires may have seemed too overwhelming for them. If this was the case, then there will be inaccuracies in their scores. Additionally, the sample is very small, and thus, it may be hard to generalize to the population.

Other potential factors that were not included on the list may also affect academic motivation, including the understanding of their major and why they chose it. Future interviews can use semi-structured methods to discover more probable affecting factors related to academic motivation.

### Conclusion

Our survey study demonstrates the academic motivation of senior students from both general and specialized rehabilitation curricula in China. The response rate in our study (73.42%) is considerably higher than the average rate achieved by most electronic surveys (32.6%) [41]. Senior rehabilitation science students in specialized curricula have higher levels of academic motivation than those in general curricula. Furthermore, there is no significant difference found in the academic motivation scores based on students’ gender, specific interests, or average parental education levels. Administrators and experts in the field of education should make efforts to promote specialized education and deliberate about how to draw on the experience of the different educational structures in their own context.

## Supplementary Information

**Additional file 1.**

## Data Availability

Data and other relevant material will be provided for noncommercial use by the corresponding author.

## Abbreviations

AMS: The Academic Motivation Scale
WFOT: World Federation of Occupational Therapists
WCPT: World Confederation for Physical Therapy
GPA: Grade point average
PT: Physical therapy
OT: Occupational therapy
