# Supplementary material for: Academic motivation among senior students majoring in rehabilitation related professions in China
Source: BMC Med Educ. 2021 Nov 17;21:582. doi: 10.1186/s12909-021-03016-9 (PMC8597206; doi:10.1186/s12909-021-03016-9)
Supplement: Supplementary file 1 — Additional file 1. [file 12909_2021_3016_MOESM1_ESM.pdf]

ACADEMIC MOTIVATION SCALE (AMS-C 28)

COLLEGE (CEGEP) VERSION

Robert J. Vallerand, Luc G. Pelletier, Marc R. Blais, Nathalie M. Brière,
Caroline B. Senécal, Évelyne F. Vallières, 1992-1993

Educational and Psychological Measurement, vols. 52 and 53

Scale Description

This scale assesses the same 7 constructs as the Motivation scale toward College (CEGEP) studies. It contains 28 items assessed on a 7-point scale.

References

Vallerand, R.J., Blais, M.R., Brière, N.M., & Pelletier, L.G. (1989). Construction et validation de l'Échelle de Motivation en Éducation (EME). Revue canadienne des sciences du comportement, 21, 323-349.

WHY DO YOU GO TO COLLEGE (CEGEP) ?

Using the scale below, indicate to what extent each of the following items presently corresponds to one of the reasons why you go to college (CEGEP).

Does not correspond at all		Corresponds a little		Corresponds moderately		Corresponds a lot		Corresponds exactly
**1**		**2**	**3**	**4**	**5**		**6**	**7**

WHY DO YOU GO TO COLLEGE (CEGEP) ?

1. Because with only a high-school degree I would not find a high-paying job later on.	1	2	3	4	5	6	7
2. Because I experience pleasure and satisfaction while learning new things.	1	2	3	4	5	6	7
3. Because I think that a college (CEGEP) education will help me better prepare for the career I have chosen.	1	2	3	4	5	6	7
4. For the intense feelings I experience when I am communicating my own ideas to others.	1	2	3	4	5	6	7
5. Honestly, I don't know; I really feel that I am wasting my time in school.	1	2	3	4	5	6	7
6. For the pleasure I experience while surpassing myself in my studies.	1	2	3	4	5	6	7
7. To prove to myself that I am capable of completing my college (CEGEP) degree.	1	2	3	4	5	6	7
8. In order to obtain a more prestigious job later on.	1	2	3	4	5	6	7
9. For the pleasure I experience when I discover new things never seen before.	1	2	3	4	5	6	7
10. Because eventually it will enable me to enter the job market in a field that I like.	1	2	3	4	5	6	7
11. For the pleasure that I experience when I read interesting authors.	1	2	3	4	5	6	7
12. I once had good reasons for going to college (CEGEP); however, now I wonder whether I should continue.	1	2	3	4	5	6	7
13. For the pleasure that I experience while I am surpassing myself in one of my personal accomplishments.	1	2	3	4	5	6	7
14. Because of the fact that when I succeed in college (CEGEP) I feel important.	1	2	3	4	5	6	7
15. Because I want to have "the good life" later on.	1	2	3	4	5	6	7

Does not correspond at all		Corresponds a little		Corresponds moderately		Corresponds a lot		Corresponds exactly
1		2	3	4	5		6	7

WHY DO YOU GO TO COLLEGE (CEGEP) ?

16. For the pleasure that I experience in broadening my knowledge about subjects which appeal to me.	1	2	3	4	5	6	7
17. Because this will help me make a better choice regarding my career orientation.	1	2	3	4	5	6	7
18. For the pleasure that I experience when I feel completely absorbed by what certain authors have written.	1	2	3	4	5	6	7
19. I can't see why I go to college (CEGEP) and frankly, I couldn't care less.	1	2	3	4	5	6	7
20. For the satisfaction I feel when I am in the process of accomplishing difficult academic activities.	1	2	3	4	5	6	7
21. To show myself that I am an intelligent person.	1	2	3	4	5	6	7
22. In order to have a better salary later on.	1	2	3	4	5	6	7
23. Because my studies allow me to continue to learn about many things that interest me.	1	2	3	4	5	6	7
24. Because I believe that a few additional years of education will improve my competence as a worker.	1	2	3	4	5	6	7
25. For the "high" feeling that I experience while reading about various interesting subjects.	1	2	3	4	5	6	7
26. I don't know; I can't understand what I am doing in school.	1	2	3	4	5	6	7
27. Because college (CEGEP) allows me to experience a personal satisfaction in my quest for excellence in my studies.	1	2	3	4	5	6	7
28. Because I want to show myself that I can succeed in my studies.	1	2	3	4	5	6	7

© ***Robert J. Vallerand, Luc G. Pelletier, Marc R. Blais, Nathalie M. Brière, Caroline B. Senécal, Évelyne F. Vallières, 1992***

KEY FOR AMS-28

# 2, 9, 16, 23	Intrinsic motivation - to know
# 6, 13, 20, 27	Intrinsic motivation - toward accomplishment
# 4, 11, 18, 25	Intrinsic motivation - to experience stimulation
# 3, 10, 17, 24	Extrinsic motivation - identified
# 7, 14, 21, 28	Extrinsic motivation - introjected
# 1, 8, 15, 22	Extrinsic motivation - external regulation
# 5, 12, 19, 26	Amotivation
